# Role of sputum examination for acid fast bacilli in tuberculous pleural effusion

**DOI:** 10.4103/0970-2113.76296

**Published:** 2011

**Authors:** Arunabha Datta Chaudhuri, Sourin Bhuniya, Sudipta Pandit, Atin Dey, Subhasis Mukherjee, Pulakesh Bhanja

**Affiliations:** *Department of Chest Medicine, R. G. Kar Medical College, Kolkata, West Bengal, India*; 1 *Department of Chest Medicine, Calcutta National Medical College, Kolkata, West Bengal, India*

**Keywords:** *Mycobacterium tuberculosis*, pleural biopsy, pleural effusion, sputum examination

## Abstract

**Background:**

Sputum for acid fast bacilli (AFB) is seldom looked for in the etiological diagnosis of tuberculous pleural effusion usually due to the absence of any parenchymal lesion radiologically, but presence of tubercle bacilli in sputum may have important epidemiological and therapeutic implication.

**Aims:**

This study aims to evaluate the role of sputum examination for AFB in the patients of tuberculous pleural effusion with no apparent lung parenchymal lesion radiologically.

**Settings and Design:**

Forty-five consecutive indoor patients of suspected tuberculous pleural effusion having no apparent lung parenchymal lesion on chest radiography were selected for our study. It was a prospective and observational study conducted over a period of 1 year.

**Materials and Methods:**

After confirming the etiology of pleural effusion as tuberculous by biochemical, cytological, histopahtological, and microbiological tests, emphasis was given on sputum examination for AFB by smear examination and culture for *Mycobacterium tuberculosis*.

**Results:**

Sputum was bacteriologically (smear and /or culture) positive for tuberculosis in 10 out of 30 cases (33.33%) in which tuberculous etiology was confirmed by histology and /or bacteriology (definite tuberculosis). No sputum AFB (smear and culture) was found in 15 cases of probable tuberculosis where tuberculous etiology was established by indirect methods like Adenosine de aminase level more than 40 unit/l and other relevant investigations. Over all, sputum was bacteriologically smear and/or culture positive in 10 out of 45 cases (22.22%).

**Conclusion:**

Careful and thorough sputum examination in cases of tuberculous pleural effusion may help as a diagnostic tool and it has therapeutic and epidemiological implications.

## INTRODUCTION

Parenchymal lung lesions are usually not apparent on chest radiography in cases of tuberculous pleural effusion. Sputum for acid fast bacilli (AFB) is seldom looked for in such cases. However, with the use of computed tomography parenchymal lesion including focal areas of subpleural cavitation and lymphadenopathy can be visualized which is not apparent on routine chest radiography.[1] This was also found at autopsy and in pneumonectomy specimens at surgery before the advent of CT scan.[2] Therefore, in such cases there is possibility of isolation of tubercle bacilli in sputum which help us as a diagnostic tool. Moreover, presence of tubercle bacilli in sputum may have epidemiological significance, and it demands thorough examination of all contacts of tuberculous pleural effusion cases and has significant therapeutic implication also. Therefore, we carried out this study to evaluate the role of sputum examination for AFB in patients of tubercular pleural effusion in a tertiary hospital of Kolkata, where it is estimated that the prevalence of tuberculosis (TB) is around 185 cases per one lac population per year and those with human immunodeficiency virus (HIV) coinfection is around 5% of the TB cases.

## MATERIALS AND METHODS

The study was done in a tertiary hospital of Kolkata over a period of 1 year. Forty-five consecutive cases of suspected tuberculous pleural effusion with no apparent lung parenchymal lesion on chest radiography were evaluated by thoracentesis. Subsequently biochemical (protein, sugar, lactate dehydrogenase, Adenosine de aminase (ADA)), cytological (cell type, cell count, malignant cell) and microbiological (Gram’s stain, Ziehl–Neelsen stain for acid fast bacilli and culture for mycobacteria tuberculosis by conventional Lowenstein–Jensen (L–J) method) tests for pleural fluid were done. Centrifuged deposits were used for smear examination for AFB. Biochemical parameters of pleural fluid were determined using a selective discrete multichannel analyzer. Total protein concentration (g/dl) was measured by auto analyzer using Biuret method (Technicon RA 1000),[3] lactate dehydrogenase (LDH) level were measured by auto analyzer using modified International Federation of Clinical Chemistry (IFCC) method (Technicon RA1000)[3] and ADA levels measured by auto analyzer using the colorimetric end point method described by Guisti and Galanti[4] (Microlab 300, Merck, The Netherlands). Then after obtaining proper informed consent pleural biopsy was done in all patients using Abram’s pleural biopsy needle. The biopsy specimens were sent for histopathological and microbiological (Z–N stain) examination for tuberculosis. Cultures of pleural biopsy specimen for tubercle bacilli were done by conventional L–J method.

After history taking, clinical examination and relevant investigation, pleural effusion were considered to be of definite tuberculous etiology if centrifuged pleural fluid or pleural tissue obtained by biopsy was positive for AFB by Z–N staining, pleural fluid or pleural tissue was culture positive for tubercle bacilli by L–J method and/or pleural tissue specimen revealed typical epitheloid granuloma consistent for tuberculosis.[5] In those cases where there were no histopathological and/or microbiological evidences of tuberculosis but clinical examination, relevant investigations, pleural fluid ADA level >40 U/l[6] and after excluding other causes were considered to be of probable tuberculous etiology. Test for HIV was done only in high risk cases because the hospital is situated in the low prevalent zone of HIV infection.

After confirming the etiology of pleural effusion as tuberculous by histology and/or bacteriology or by indirect methods like pleural fluid ADA level (probable tuberculosis), emphasis was given on meticulous examination sputum smear for AFB by Z–N method and sputum culture for *Mycobacterium tuberculosis* by conventional LJ method. Spontaneous morning sputa were collected under direct supervision on three consecutive days for smear examinations and for sputum culture one spontaneous morning sample was collected under supervision. Those cases having no apparent radiological lung parenchymal lesion were selected and none was subjected to any specific antitubercular drug therapy prior to the study. The study was carried out after approval from the ethical committee.

## RESULTS

In our study most of the cases (55.56%) were in the 21–30 years of age group and there was no case of tuberculous pleural effusion above 60 years of age. In our series of 45 cases of pleural effusion, 29 were male (64.44%) and 16 (35.56%) were female. It is evident from our study that the incidence of tuberculous pleural effusion was more in urban than in rural population. History of contact was obtained in 15 cases (33.33%), of this 10 were males and 5 were females. No case in this study was found to be HIV positive.

Analysis of pleural fluid showed it was mostly straw colored (97.77%) and in only one out 45 cases it was hemorrhagic. In this study white blood cell count in pleural fluid was in the range of 1000–3000/cumm in 60% cases and the cell type in pleural fluid were predominantly lymphocytes (95.45%). Malignant cell was not found in pleural fluid in any case. Light’s criteria[6] were used to distinguish exudates from transudate and in our study pleural fluid was exudate in all cases.

In our study AFB were found by Z–N staining method of centrifuged deposit of plural fluid in 4 out of 45 cases (8.88%). No bacteria could be detected from the smear of pleural fluid stained by Gram’s method in each case. Nine out of 45 cases (20%) had growth of *Mycobacterium tuberculosis* on culture of pleural fluid which was later confirmed by subculture. Pleural tissue was obtained by pleural biopsy in 39 of 45 cases (86.66%). In six cases, no pleural tissue were obtained even after repeat biopsy. Twenty-three cases (51.11%) showed caseating epitheloid granuloma with giant cell, 16 cases showed picture of chronic nonspecific inflammation on histopathological examination. Examination of pleural tissue for AFB by Z–N method was done in 37 cases as out of 39 cases, in two cases amount of pleural tissue were very scanty. In six out of 37 cases (16.21%) AFB was found in pleural tissue smear. Twenty one out of 37 (56.75%) cases showed growth of *Mycobacterium tuberculosis* on culture of pleural tissue in L–J media. In our study pleural tissue culture was positive in 21 cases. In one case pleural tissue smear was positive only but all other bacteriological examination was negative. In two cases pleural fluid culture was positive only but all other bacteriological test was negative. Therefore, 24 cases were diagnosed bacteriologically as tuberculous.

Tuberculous etiology of pleural effusion was diagnosed by histology in 23 out of 45 cases and by bacteriology in 24 out of 45 cases. Both histology and bacteriology was positive in 17 cases, in six cases histology were positive but bacteriological examination were negative, and in seven cases bacteriological examination were positive but histology were negative. Therefore, in 30 cases, tuberculous etiology of pleural effusion was confirmed by histology and/or bacteriology (definite tuberculosis). In remaining 15 cases (probable tuberculosis), diagnosis of tubrculous etiology was established by aspiration of straw colored, exudative lymphocytic predominant pleural fluid with ADA level more than 40 IU/l and excluding other causes of pleural effusion [Table 1].

**Table 1:** Diagnosis of tuberculous pleural effusion by different methods (*n*=45)

Histology	Bacteriology	Histology positive but bacteriology negative	Bacteriology positive but histology negative	Both histology and bacteriology positive	Histology and/or bacteriology	Other methods including ADA
23	24	6	7	17	30	15
(51.11)	(53.33)	(13.33)	(15.56)	(37.77)	(66.66)	(33.34)

ADA: Adenosine de aminase; Figures in parentheses are in percentage

After confirming etiology of pleural effusion as tuberculous, examination of sputum for AFB by Z–N method and culture in L–J media were done in all 45 cases. In our study, in five out of 45 cases (11.11%) AFB was found in the sputum smears. Among 45 cases, 10 cases (22.22%) showed growth of *Mycobacterium tuberculosis* on culture in L–J media which was later confirmed by sub culture [Table 2]. Out of 45 cases, sputum smear for AFB were positive in five cases, sputum culture were positive in 10 cases, both smear and culture were positive in five cases and in five cases sputum smear were negative but culture were positive. In the remaining 35 cases, both culture and smear were negative. Comparison of the sputum smear and culture reports between the two groups (definite and probable tuberculosis) revealed positive sputum smears in five cases (16.66%), positive sputum culture in 10 cases (33.33%), positive sputum smear and/or culture in 10 cases (33.33%) in the definite tuberculosis group, whereas sputum smear and culture were negative in all the cases of probable tuberculosis [Table 2].

**Table 2:** Results of sputum examination among patients with tuberculous pleural effusion cases diagnosed by different methods

Methods of etiological diagnosis	Number of cases	Sputum smear positive	Sputum culture positive	Sputum bacteriologically positive (smear and/or culture)
Diagnosed by histology	23	4 (17.39)	8 (34.78)	8 (34.78)
Diagnosed by bacteriology	24	5 (20.83)	9 (37.55)	9 (37.5)
Diagnosed by histology and/or bacteriology (definite tuberculosis)	30	5 (16.66)	10 (33.33)	10 (33.33)
Diagnosed by other methods (probable tuberculosis)	15	0	0	0
Total number of cases	45	5 (11.11)	10 (22.22)	10 (22.22)

Figures in parentheses are in percentage

## DISCUSSION

Most of the cases were in the age group of 21–30 in our study. In general, patients with tuberculous pleural effusion are younger than patients with parenchymal tuberculosis.[6] The findings in our study that the incidence of tuberculous pleural effusion is more in urban then in rural population might be due to the fact the greater number of urban population attended and got admitted in our city based tertiary hospital due to easy accessibility of the hospital. Lymphocyte predominance with lymphocyte count greater than 50% in pleural fluid was observed in our study in 95.45% cases. It has been reported that in most patients of tuberculous pleural effusion, pleural fluid differential count reveals small lymphocyte more than 50%.[6] In 4 out of 45 cases (8.88%) AFB was found in smear of pleural fluid. Antoniskis *et al*.,[7] found AFB in pleural fluid smear in 7% cases of tuberculous pleural effusion with no apparent radiological parenchymal infiltrate and in 27% cases of tuberculous pleural effusion having pulmonary infiltrate. Ghosal *et al*.[8] found AFB in pleural fluid smear in two out of 30 cases (6.66%) of tuberculous pleural effusion with no parenchymal lesion on chest X-ray. Culture of pleural fluid showed growth of *Mycobacterium tuberculosis* in 9 out of 45 cases (20%) in our study, which is similar to that of Salazar *et al*.,[9] (19%). Many other studies have shown positive pleural fluid culture for *Mycobacterium tuberculosis* in <30% cases.[10–12] Culture has been done by conventional method in our study. Use of a Bactec system with bed side inoculation provides better yield and faster result than conventional method.[6]

AFB were found in smear of pleural tissue in 16.21% cases in our study which is comparable to that of Bueno, *et al*.[13] (10%) and Antoniskis, *et al*.[7] (17%). The yield of culture positive pleural tissue (56.75%) in this study is comparable with those of Gopi *et al*.[14] (39%–80%) and Valdes *et al*.[15] (56%). In our study pleural biopsy material on histology showed evidence of Tuberculosis in 51.11% of cases which is within the range (50%–97%) found by Gopi *et al*.[14] and pleural biopsy tissue was culture positive in 56.75% of cases. So, culture of pleural biopsy specimen is as useful a diagnostic tool as histological examination. Moreover, in four cases histological evidence of tuberculosis was negative but culture was positive. Thus failure to find granuloma on pleural tissue does not exclude diagnosis of tuberculosis and culture of tissue may be positive when histology is negative.

In five out of 45 (11.11%) cases AFB were found in sputum smear which is comparable with Ghosal *et al*.[8] (10%). In our study culture of sputum showed growth of *Mycobacterium tuberculosis* in 10 out of 45 cases (22.22%). Antonskis *et al*.[7] evaluated 59 confirm cases of tuberculous pleural effusion of which 27 patients had radiological parenchymal infiltration and 32 patients had no parenchymal infiltration. Sputum for mycobacterium culture was positive in 60% and 23% cases, respectively. Conde *et al*.[16] prospectively evaluated the diagnostic yield of AFB smear and culture in 84 patients with tuberculous pleural effusion and they were positive in 44 patients (52%). Sputum smears were positive in 10 patients and cultures were positive in all 44 cases. Sputum smears were positive in three patients and culture became positive in 11 cases (27%) out of 30 diagnosed case of tuberculous pleural effusion having no apparent lung parenchymal lesion on routine chest radiography as described by Ghosal *et al*.[8]

It is worth mentioning here that in our study tuberculous etiology could not be definitely established either by histology or bacteriology in 15 cases but we considered them as probable tuberculosis on the basis of clinical suspicion, pleural fluid cytology and biochemistry including ADA and ruling out other possible etiology. Both sputum smear and culture were negative in those 15 cases. The possible explanation is that parenchymal involvement may be negligible or they may represent pleural effusion of nonspecific etiology. So, there is a scope of further evaluation of indirect methods of diagnosis of tuberculous pleural effusion.

In tuberculous pleural effusion routine posterio anterior (PA) and lateral radiography usually do not reveal any parenchymal lesion as opacity of pleural effusion conceals parenchymal lesion even if they are present. Berger *et al*.[17] found that 18 out of 49 patients (37%) with proved tuberculous pleural effusion had coexistent active parenchymal disease on chest radiography. Pneumonectomy specimens at surgery had confirmed that parenchymal lesion was present even in those patients having no radiologically apparent parenchymal lesion.[2] CT scan of thorax can detect focal areas of subpleural cavitation unappreciated on radiography and/or confirmed lymphadenopathy that otherwise goes undetected.[1] Prior attention for sputum smear examination and culture has not been given due to the absence of apparent radiological lung parenchymal lesion in tuberculous pleural effusion. But with the changing scenario after advent of CT scan, sputum examination is relevant and our study also proves that.

From the sputum smear and culture examination results of our study it is evident that all patients of tuberculous pleural effusion even with no radiological lung parenchymal lesion need meticulous sputum examination for AFB smear and culture. Therefore, careful and thorough sputum examination may help as a diagnostic tool.

Moreover, due to absence of apparent lung parenchymal lesion radiologically, patients are usually considered sputum negative and noninfectious. However, presence of tubercle bacilli in sputum may be epidemiologically significant and it needs thorough contact examination of all cases of tuberculous pleural effusion including those having no apparent parenchymal lesion on chest X-ray.

Besides, all cases tuberculous pleural effusion are considered extra-pulmonary and in these cases routine sputum examination for AFB is not done and they are treated with category III regimen under Revised National Tuberculosis Programme (RNTCP), but presence of sputum positivity in some patients of tuberculous pleural effusion should make therapeutic approach different and those cases should be considered for category I regimen. Therefore, thorough sputum examination has significant therapeutic implication as well.

## CONCLUSION

Examination of sputum for AFB smear in all patients of tuberculous pleural effusion is simple and inexpensive and has a significant therapeutic and epidemiological importance.
